# Asociación entre la 25-hidroxivitamina D y el antígeno prostático específico: un estudio retrospectivo en hombres sin patologías prostáticas

**DOI:** 10.1515/almed-2023-0157

**Published:** 2023-12-04

**Authors:** Javier Laguna, Robin Wijngaard, Susana Hidalgo, Cristina González-Escribano, Victoria Ortiz, José Luis Bedini, Xavier Filella

**Affiliations:** Servicio de Bioquímica y Genética Molecular, CDB, Hospital Clínic de Barcelona, Barcelona, España; Laboratorio CORE, CDB, Hospital Clínic de Barcelona, Barcelona España

**Keywords:** 25-hidroxivitamina D, cáncer de próstata, antígeno prostático específico, vitamina D

## Abstract

**Objetivos:**

Aunque estudios recientes asocian la vitamina D con el cáncer de próstata, otros estudios descartan una asociación entre esta vitamina y el cáncer de próstata o el antígeno prostático específico (PSA). Dado que no se pueden extraer conclusiones de los datos existentes, realizamos un estudio para analizar la relación entre el PSA y la 25-hidroxivitamina D [25(OH)D].

**Métodos:**

Un total de 415 sujetos sin patologías prostáticas fueron seleccionados, y se clasificaron por edad y concentraciones de 25(OH)D. El análisis estadístico se realizó con la prueba de Shapiro-Wilk, la prueba *t* de Student, ANOVA, y el coeficiente de correlación de Pearson. Además, se calculó el tamaño mínimo de muestra requerido para obtener resultados estadísticamente significativos en función de la concentración de 25(OH)D. Así mismo, se realizó la prueba *t* de Student para muestras pareadas para analizar a los individuos con dos determinaciones de PSA espaciadas en el tiempo en los que las concentraciones de 25(OH)D aumentaron o disminuyeron más de un 25 %.

**Resultados:**

Observamos una leve correlación entre la edad y el PSA (r=0,379, p<0,001). Sin embargo, al comparar la concentración de PSA entre grupos en función de 25(OH)D, no se hallaron diferencias significativas (p=0,891): 1,25±1,32 μg/L (grupo con 25(OH)D<50 nmol/L) y 1,17±0,90 (grupo con 25(OH)D≥50 nmol/L). El coeficiente de correlación de Pearson fue casi 0. El tamaño mínimo de la muestra necesario para obtener resultados estadísticamente significativos fue de 815.346 hombres. No observamos diferencias en las concentraciones de PSA en los individuos que se sometieron a dos determinaciones.

**Conclusiones:**

Nuestros resultados muestran que no existe asociación entre los niveles de 25(OH)D y de PSA en hombres sin patologías prostáticas.

## Introducción

La vitamina D es una vitamina liposoluble que se puede obtener a través de los alimentos (vitamina D2) o de la transformación cutánea del 7-deshidrocolesterol en presencia de radiación ultravioleta (vitamina D3). Independientemente de la fuente, se metaboliza a 25-hidroxivitamina D [25(OH)D] y, posteriormente, a la forma biológicamente activa, calcitriol o 1,25-dihidroxivitamina D [1,25(OH)_2_D]. La hormona activa se une al receptor de la vitamina D (VDR) para regular el metabolismo del calcio y del fósforo, lo que es esencial para la remodelación ósea, la reabsorción del calcio en el túbulo distal y la absorción intestinal del calcio y el fósforo [1, 2]. Aunque 1,25(OH)_2_D es la hormona activa, la concentración sérica de 25(OH)D es el parámetro analítico que se utiliza habitualmente para evaluar el estado de vitamina D [3].

El déficit de vitamina D se ha asociado con un mayor riesgo de desarrollar distintos tipos de cáncer, incluido el cáncer de próstata [4]. Según la Agencia Internacional para la Investigación del Cáncer (*International Agency for Research on Cancer*, IARC), en 2020, 1,41 millones de personas fueron diagnosticados de cáncer de próstata en todo el mundo, lo que representa alrededor de un 7 % de todos los casos de cáncer diagnosticados en hombres. Además, el cáncer de próstata es la segunda causa de muerte por cáncer en hombres en todo el mundo, habiendo provocado unas 375.000 muertes en 2020. Cabe señalar que la incidencia del cáncer de próstata varía con la edad de la población. El riesgo de desarrollar cáncer de próstata se incrementa con la edad, siendo más frecuente en hombres mayores de 50 años [5].

El primer estudio realizado para investigar la relación entre el déficit de vitamina D y el cáncer de próstata se publicó a finales del siglo XX, mostrando un posible efecto protector de la vitamina D frente al cáncer de próstata [6]. Las guías clínicas de EAU-EANM-ESTRO-ESUR-ISUP-SIOG sobre el cáncer de próstata señalan una asociación en forma de U, estando asociadas tanto las concentraciones bajas como las altas con un mayor riesgo de desarrollar cáncer de próstata [7].

Recientemente, un estudio realizado en células de cáncer de próstata ha propuesto que la vitamina D actúa como regulador del metabolismo [8]. La testosterona y su metabolito 5α-reducido, la dihidrotestosterona (DHT), se une al receptor androgénico (RA), un factor de transcripción nuclear dependiente de ligando, que provoca el crecimiento tumoral y una mayor expresión del antígeno prostático específico (PSA), entre otras funciones [9]. Por esta razón, el objetivo de la terapia de privación de andrógenos (ADT) es reducir las concentraciones de testosterona y DHT. Sin embargo, en algunos casos, el cáncer deja de responder a la ADT y progresa a una forma más agresiva (cáncer de próstata resistente a la castración) [10]. Smith y col [8]. observaron que la vitamina D inhibe la conversión intracrina de la dehidroepiandrosterona (DHEA) en testosterona (y DHT). Ante la ausencia de un ligando para el RA, el tumor no crecería, y la concentración de PSA disminuiría. Este hallazgo sería interesante para el tratamiento de la enfermedad, especialmente, para la forma resistente a la castración.

Estudios posteriores refuerzan la hipótesis de que las concentraciones de vitamina D están relacionadas no solo con el riesgo de desarrollar cáncer de próstata, sino también otros tipos de cáncer [11], [12], [13], [14]. Sin embargo, otros estudios contradicen esta hipótesis y no muestran que exista relación entre la vitamina D, el cáncer de próstata y las concentraciones de PSA [15], [16], [17], [18], por lo que no se pueden extraer conclusiones definitivas de las evidencias existentes. Por esta razón, nuestro objetivo fue evaluar la posible relación entre el PSA y la vitamina D, a través de la determinación de 25(OH)D.

## Materiales y métodos

### Sujetos

Se realizó un estudio retrospectivo que incluyó 415 hombres atendidos en las consultas externas de nuestro hospital entre enero de 2015 y marzo de 2021. Los sujetos elegibles para el estudio fueron hombre mayores de 18 años (rango de edad: 34–88 años). Se excluyó a aquellos pacientes con patología prostática (antecedentes de cáncer de próstata, prostatitis e hiperplasia benigna de próstata), insuficiencia renal (creatinina sérica>0,11 mmol/L) y/o insuficiencia hepática (alanina aminotransferasa, aspartato aminotransferasa o gamma-glutamil transferasa>40 U/L; o fosfatasa alcalina>116 U/L). Durante el periodo de estudio, el 22 % de los pacientes tomaba diariamente suplementos de vitamina D.

El estudio se llevó a cabo de acuerdo con los principios de la Declaración de Helsinki (revisada en 2013) y fue aprobado por el Comité de Ética de Investigación de nuestro hospital (Reg. HCB/2021/0631).

### Determinación de PSA y 25(OH)D

Las muestras de sangre se recogieron entre las 8 y las 10 de la mañana en tubos BD Vacutainer^®^ de 5 mL (que contenían un activador de coagulación y gel separador). Teniendo en cuenta las oscilaciones estacionales, se extrajeron muestras en todas las estaciones del año.

El PSA sérico se midió mediante dos inmunoensayos quimioluminiscentes. Entre 2015 y 2019, se midió en un analizador ADVIA Centaur^®^ XP (Siemens Healthineers, Tarrytown, NY, EEUU). A partir de 2019, se utilizó el analizador Atellica^®^ IM 1600 (Siemens Healthineers, Tarrytown, NY, EEUU). La 25(OH)D sérica se midió mediante inmunoensayo quimioluminiscente en un analizador LIAISON^®^ XL (DiaSorin, Saluggia, Italia).

### Análisis estadístico

Se crearon cuatro subgrupos en función de los siguientes intervalos de edad: de 34 a 49 años, de 50 a 59 años, de 60 a 69 años, y mayores de 70 años, y se establecieron dos grupos en función de las concentraciones de 25(OH)D: <50 nmol/L y ≥50 nmol/L. El umbral de 50 nmol/L (20 μg/L) fue establecido según las recomendaciones más recientes [19]. Además, se dividió a los sujetos en diferentes grupos para analizar la asociación en forma de U, del mismo modo que los modelos de riesgos proporcionales de Cox propuestos por Kristal y col. para establecer los índices de riesgo para la asociación entre los niveles plasmáticos de vitamina D y el riesgo de desarrollar cáncer de próstata, incluyendo covariantes como el índice de masa corporal, los antecedentes de diabetes, y los antecedentes familiares de cáncer de próstata [14].

Se calcularon las medias y desviaciones estándar de las concentraciones de PSA. La normalidad de los datos se analizó mediante la prueba de Shapiro-Wilk y, en caso necesario, se realizaron transformaciones logarítmicas. Las diferencias entre variables con distribución normal se analizaron mediante la prueba *t* de Student y ANOVA. Se realizó un modelo de regresión logística multivariante para analizar las principales variables en estudio (concentraciones de PSA y edad) y el resultado clínico (concentraciones bajas o normales de vitamina D, considerándolas como una variable binaria y aplicando un umbral de 50 nmol/L).

Así mismo, analizamos la correlación entre las variables calculando los coeficientes de correlación de Pearson. Además, en el estudio de la relación entre el PSA y 25(OH)D, calculamos el tamaño mínimo de la muestra necesario para obtener diferencias estadísticamente significativas entre los grupos, en función de nuestros resultados, con una potencia estadística de 0,80. Empleamos la prueba t de Student para muestras relacionadas para analizar los cambios en las concentraciones de PSA, en individuos con dos determinaciones de PSA y 25(OH)D espaciadas en el tiempo (con una diferencia mínima de seis meses entre mediciones) en los que aumentó (52 sujetos) o disminuyó (22 sujetos) la 25(OH)D más de un 25 %.

Todos los análisis estadísticos se realizaron con el *software*R (versión 4.1.3). Valores de p<0,05 se consideraron estadísticamente significativos.

## Resultados

En la Tabla 1 se muestran las concentraciones de PSA y 25(OH)D, en función de la edad de los individuos. Observamos diferencias significativas en las concentraciones de PSA dependientes de la edad, clasificada por grupos etarios (p<0,001), pero no en las concentraciones de 25(OH)D (p=0,409). La comparación por pares realizada aplicando la prueba *t* de Student reveló mayores concentraciones de PSA en los hombres de entre 60 y 69 años y en hombres mayores de 70 años, que en los grupos de hombres más jóvenes. Observamos una leve correlación entre la edad y la concentración de PSA, con un r=0,379 (p<0,001).

**Tabla 1:** Media y desviaciones estándar (DE) de las concentraciones de PSA y 25(OH)D en función de la edad.

Grupo	n	Edad (media±DE), años	25(OH)D (media±DE), nmol/L	PSA concentration (mean±SD), μg/L
34–49	72	44±4	23*,*8±10*,*3	0*,*67±0*,*35
50–59	144	55±3	21*,*7±9*,*3	0*,*97±0*,*79
60–69	137	64±3	22*,*9±10*,*0	1*,*50±1*,*32
≥70	62	74±4	24*,*8±10*,*0	1*,*72±1*,*38

25(OH)D, 25-hidroxivitamina D; PSA, antígeno específico.

Se observó deficiencia (<30 nmol/L) e insuficiencia (30–50 nmol/L) de vitamina D en 185 de los 415 hombres (45 %), con una concentración media de 25(OH)D de 36,4±8,7 nmol/L (rango: 10,2–49,4 nmol/L). En el resto de individuos, las concentraciones de 25(OH)D fueron ≥50 nmol/L (rango: 50,0–158,9 nmol/L). No se observaron diferencias estadísticas en relación a la edad entre los grupos (<50 nmol/L: 59±10 años y ≥50 nmol/L: 59±11 años; p=0,920). El modelo de regresión logística multivariante no mostró una asociación significativa entre los niveles de 25(OH)D y la edad (p=0,239) o los niveles de PSA (p=0,581).

No se hallaron diferencias significativas en las concentraciones de PSA entre los dos grupos en función del valor umbral de 25(OH)D de 50 nmol/L (1,25±1,32 μg/L y 1,17±0,90 μg/L, respectivamente, p=0,891, Tabla 2). Además, tampoco se observaron diferencias significativas en las concentraciones de PSA entre los hombres con deficiencia e insuficiencia de 25(OH)D (1,25±1,63 μg/L y 1,25±1,19 μg/L, respectivamente, p=0,350). Con respecto al estudio de la relación en forma de U según los umbrales de los modelos propuestos por Kristal y col. [14], no se observó una relación en forma de U entre las concentraciones de vitamina D y de PSA (Tabla 2).

**Tabla 2:** Medias y desviaciones estándar (DE) de las concentraciones de PSA en función de la concentración de 25(OH)D.

	Grupo	n	Concentración de 25(OH)D (media±DE), nmol/L	Concentración de PSA (media±DE), μg/L	Valor p
Según las últimas recomendaciones [19]	<50	185	36*,*4±8*,*7	1*,*25±1*,*32	0*,*891
	≥50	230	73*,*9±20*,*0	1*,*17±0*,*90	
Modelos 1 y 2 de Kristal y col. [14]	<37*,*5	89	28*,*7±5*,*7	1*,*32±1*,*63	0*,*955
	37*,*5–50	96	43*,*7±3*,*5	1*,*19±0*,*96	
	50–75	142	60*,*9±6*,*7	1*,*21±0*,*95	
	≥75	88	95*,*1±15*,*7	1*,*09±0*,*80	
Modelo 3 de Kristal y col. [14]	<44*,*1	141	33*,*1±7*,*7	1*,*21±1*,*37	0*,*972
	44*,*1–58*,*2	98	50*,*7±4*,*0	1*,*23±1*,*08	
	58*,*2–72*,*9	84	64*,*6±4*,*2	1*,*24±0*,*93	
	72*,*9–90*,*7	41	81*,*4±4*,*5	1*,*13±0*,*80	
	≥90*,*7	51	104*,*6±14*,*5	1*,*11±0*,*80	

25(OH)D, 25-hidroxivitamina D; PSA, antígeno prostático específico.

**Figura 1:**
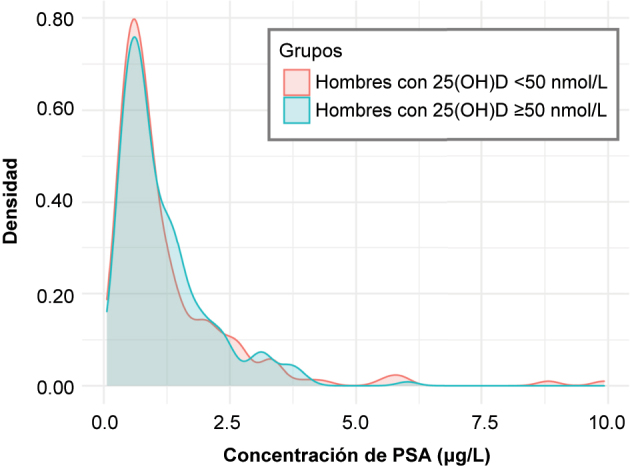
Distribución de las concentraciones de PSA en hombres en función de las concentraciones de 25(OH)D. 25(OH)D: 25-hidroxivitamina D; PSA: antígeno prostático específico

La Figura 1 muestra la distribución de las concentraciones de PSA según el grupo de 25(OH)D. Observamos que la distribución de los dos grupos casi se solapa, debido a la similitud de los datos. El coeficiente de correlación de Pearson fue próximo a 0 (r=−0,001; p=0,985), lo que indica que no existe una relación lineal entre el PSA y las concentraciones de 25(OH)D.

Se calculó el tamaño mínimo de la muestra necesario para obtener diferencias estadísticamente significativas entre los sujetos con 25(OH)D<50 nmol/L y aquellos con 25(OH)D≥50 nmol/L. Se obtuvo un valor *d* de Cohen de 0,004, lo que indica un tamaño del efecto muy pequeño entre los grupos de 25(OH)D. El cálculo del tamaño muestral con una potencia de 0,80 mostró que era necesario incluir a 815.346 hombres en cada grupo para poder obtener diferencias estadísticamente significativas.

Así mismo, se estudiaron los cambios en las concentraciones de PSA en aquellos individuos que contaban con dos determinaciones de PSA y 25(OH)D espaciadas en el tiempo. No hallamos diferencias estadísticamente significativas en relación a las concentraciones de PSA entre las determinaciones de aquellos individuos en los que la 25(OH)D aumentó más de un 25 % (1,10±0,97 μg/L and 1,13±0,84 μg/L, p=0,178) o disminuyó más de un 25 % (0,96±1,16 μg/L y 0,98±1,25 μg/L, p=0,533).

## Discusión

El PSA es el marcador más ampliamente utilizado para la detección y el seguimiento de los pacientes con cáncer de próstata [20]. Sin embargo, el PSA no es un marcador específico del cáncer, por lo que puede hallarse elevado en otras patologías no malignas [7]. A la luz de que algunos estudios muestran la importancia de un buen estado de vitamina D para prevenir el desarrollo y progresión del cáncer de próstata [3], realizamos este estudio para analizar su relación en hombres sin patologías prostáticas ni insuficiencia hepática o renal. Partimos de la hipótesis de que la deficiencia de vitamina D podría provocar un aumento del PSA por dos razones: (1) porque las concentraciones bajas de vitamina D inducen el cáncer de próstata y, por lo tanto, en la población con concentraciones bajas de vitamina D, se encuentra un mayor número de individuos con cáncer de próstata no detectado, a diferencia de cuando la vitamina D es elevada; y (2) porque las concentraciones bajas de vitamina D aumentan los niveles de PSA.

Observamos una leve correlación entre la edad y la concentración de PSA. El grupo de edad con concentraciones más bajas de PSA fue el de los pacientes más jóvenes (entre 34 y 49 años y entre 50 y 59 años), observándose las concentraciones más elevadas en individuos de entre 60 y 69 años y con 70 años o más, lo que concuerda con los datos publicados por Oesterling y col. [21]. Por otro lado, no se observó ninguna asociación entre la edad y los niveles de 25(OH)D. Aunque se creía que los niveles de vitamina D disminuyen con la edad, los estudios demuestran que no existen diferencias significativas en los niveles de vitamina D entre personas de diferentes edades. Esto se podría deber a la inclusión de pacientes menores de 75 años en estos estudios [22]. En nuestro estudio se incluyó a hombres de entre 34 y 88 años, aunque únicamente 10 pacientes eran mayores de 75 años, lo que podría explicar la falta de asociación observada entre los niveles de 25(OH)D y la edad.

Además, no hallamos ninguna relación entre el PSA y las concentraciones de 25(OH)D en nuestra población de estudio. No se observaron diferencias estadísticamente significativas al comparar a los sujetos con deficiencia de vitamina D y aquellos con concentraciones de 25(OH)D≥50 nmol/L. Del mismo modo, Tóth y col [15]. realizaron un estudio retrospectivo en una cohorte de 5.136 hombres, no hallando diferencias en las concentraciones de PSA entre individuos con diferentes concentraciones de vitamina D. Así mismo, al evaluar el riesgo de desarrollar cáncer, no se halló ninguna asociación entre los niveles de vitamina D y el riesgo de desarrollar cáncer de próstata [17, 18]. Sin embargo, Kristal y col. observaron una asociación en forma de U entre las concentraciones de 25(OH)D y el riesgo de desarrollar cáncer de próstata. Los autores descubrieron que los individuos con concentraciones de 25(OH)D bajas o altas presentaban un mayor riesgo de desarrollar cáncer, especialmente enfermedad avanzada [14]. En contraposición, nuestro estudio no reveló diferencias significativas en las concentraciones de PSA entre los grupos. De este modo, los hombres con concentraciones de 25(OH)D bajas o altas no presentaron concentraciones de PSA superiores a las del resto de grupos.

Cabe mencionar que se incluyó a hombres con concentraciones normales de creatinina y enzimas hepáticas, un aspecto que no se ha tenido en cuenta en otros estudios. Las patologías hepáticas afectan a las concentraciones de PSA en suero, y se ha demostrado que los pacientes con cirrosis presentan menores concentraciones de PSA que los hombres sin cirrosis [23]. Además, el déficit de vitamina D es común entre los pacientes con enfermedad renal crónica o enfermedad hepática [24, 25]. Por estas razones, la inclusión de estos individuos podría llevar a conclusiones erróneas.

Partimos de la hipótesis de que la deficiencia de vitamina D provocaría un aumento de las concentraciones de PSA, ya sea como consecuencia del efecto directo de la vitamina D sobre la síntesis del PSA, o debido a la presencia de hombres con cáncer de próstata sin diagnosticar. Nuestro estudio se basó en una determinación de 25(OH)D, que refleja las reservas de vitamina D en un determinado momento, siendo esto una limitación de la mayoría de los estudios realizados en este campo. El cáncer es un proceso a largo plazo, por lo que puede resultar complicado hallar una asociación partiendo de una única medición de los niveles de vitamina D.

Sin embargo, resulta más sencillo analizar el efecto directo de la vitamina D. En el presente estudio, no se observaron diferencias en las concentraciones de PSA entre individuos con diferentes concentraciones de 25(OH)D. Además, analizamos a los hombres que se sometieron a dos mediciones de PSA y 25(OH)D espaciadas en el tiempo, con cambios significativos en las concentraciones de 25(OH)D. Estas mediciones se analizaron con la prueba *t* de Student para muestras relacionadas, no observando diferencias estadísticamente significativas entre ellas. En 2014, Chandler y col [26]. realizaron un estudio prospectivo en 105 hombres para evaluar el impacto de los suplementos de vitamina D sobre las concentraciones de PSA, distribuyendo de forma aleatoria a los individuos en cuatro grupos (placebo, 1.000, 2.000 y 4.000 UI de vitamina D) a lo largo de un periodo de tres meses. En el grupo de hombres tratados con 4.000 UI de vitamina D, las concentraciones medias de 25(OH)D basales y a los tres meses fueron de 44,4 y 118,0 nmol/L, respectivamente. Sin embargo, este aumento en la concentración de 25(OH)D no influyó en las concentraciones de PSA, que permanecieron estables (2,15 y 2,20 μg/L al inicio y a los tres meses, respectivamente). De este modo, no existen pruebas concluyentes que sugieran que los suplementos de vitamina D influyan en los niveles de PSA.

Con respecto a las limitaciones del estudio, no podemos descartar que la 1,25(OH)_2_D influya en las concentraciones de PSA, por lo que, en futuros estudios, se debería considerar medir también los niveles de 1,25(OH)_2_D. Este metabolito es el agonista activo del VDR y regula la diferenciación y proliferación celular de gran variedad de células, incluyendo las células epiteliales prostáticas [27, 28]. Por consiguiente, se creía que las concentraciones de 1,25(OH)_2_D estaban relacionadas con el riesgo de desarrollar cáncer, así como con las concentraciones de PSA [29, 30].

En conclusión, aunque otros estudios sugieran que la vitamina D influye en las concentraciones de PSA, los resultados de nuestro estudio no evidencian que exista relación alguna con los niveles de 25(OH)D en hombres sin patología prostática y sin insuficiencia hepática o renal. Además, son necesarios más estudios para corroborar la influencia de la 1,25(OH)_2_D en la concentración de PSA.
